# Transposon Insertion Sequencing in a Clinical Isolate of Legionella pneumophila Identifies Essential Genes and Determinants of Natural Transformation

**DOI:** 10.1128/JB.00548-20

**Published:** 2021-01-11

**Authors:** Léo Hardy, Pierre-Alexandre Juan, Bénédicte Coupat-Goutaland, Xavier Charpentier

**Affiliations:** a Centre International de Recherche en Infectiologie, Team Horizontal gene transfer in bacterial pathogens, Inserm, U1111, Université Claude Bernard Lyon 1, CNRS, UMR5308, École Normale Supérieure de Lyon, Université Lyon, Villeurbanne, France; Brigham and Women's Hospital/Harvard Medical School

**Keywords:** *Legionella pneumophila*, cell viability, genetic competence, genome analysis, natural transformation systems

## Abstract

Legionella pneumophila is a Gram-negative bacterium ubiquitous in freshwater environments which, if inhaled, can cause a severe pneumonia in humans. The emergence of L. pneumophila is linked to several traits selected in the environment, the acquisition of some of which involved intra- and interkingdom horizontal gene transfer events. Transposon insertion sequencing (TIS) is a powerful method to identify the genetic basis of selectable traits as well as to identify fitness determinants and essential genes, which are possible antibiotic targets. TIS has not yet been used to its full power in L. pneumophila, possibly because of the difficulty of obtaining a high-saturation transposon insertion library. Indeed, we found that isolates of sequence type 1 (ST1), which includes the commonly used laboratory strains, are poorly permissive to saturating mutagenesis by conjugation-mediated transposon delivery. In contrast, we obtained high-saturation libraries in non-ST1 clinical isolates, offering the prospect of using TIS on unaltered L. pneumophila strains. Focusing on one of them, we then used TIS to identify essential genes in L. pneumophila. We also revealed that TIS could be used to identify genes controlling vertical transmission of mobile genetic elements. We then applied TIS to identify all the genes required for L. pneumophila to develop competence and undergo natural transformation, defining the set of major and minor type IV pilins that are engaged in DNA uptake. This work paves the way for the functional exploration of the L. pneumophila genome by TIS and the identification of the genetic basis of other life traits of this species.

**IMPORTANCE** Legionella pneumophila is the etiologic agent of a severe form of nosocomial and community-acquired pneumonia in humans. The environmental life traits of L. pneumophila are essential to its ability to accidentally infect humans. A comprehensive identification of their genetic basis could be obtained through the use of transposon insertion sequencing. However, this powerful approach had not been fully implemented in L. pneumophila. Here, we describe the successful implementation of the transposon-sequencing approach in a clinical isolate of L. pneumophila. We identify essential genes, potential drug targets, and genes required for horizontal gene transfer by natural transformation. This work represents an important step toward identifying the genetic basis of the many life traits of this environmental and pathogenic species.

## Abstract (precis)

Legionella pneumophila is the etiologic agent of a severe form of nosocomial and community-acquired pneumonia in humans. The environmental life traits of L. pneumophila are essential to its ability to accidentally infect humans.

## INTRODUCTION

Legionella pneumophila is a Gram-negative bacterium, ubiquitous in freshwater environments, where it can be found in planktonic form, in biofilm communities, or associated with amoebic protozoa which constitute its natural hosts (1). L. pneumophila can resist predation by amoebae and even establish an intracellular vacuole in which it can multiply while being protected from external environment (2). Man-made water systems have offered a new breeding ground for the development of L. pneumophila. Inhalation by humans of aerosols produced by these systems and contaminated by L. pneumophila can cause Legionnaires' disease (3). This community-acquired disease, which is most often characterized by a severe pneumonia, occurs when L. pneumophila infects alveolar macrophages (4). In both macrophages and its natural amoebic hosts, L. pneumophila replicates intracellularly by hijacking the host cellular machinery (5). This requires the Icm/Dot type IV system (6, 7), a conjugative system that can secrete more than 300 different effector proteins (8, 9). The genome of the endemic strain Paris provided early evidence of genes encoding proteins like those of eukaryotes (10), and eukaryotic-like proteins encoded by the Philadelphia-1 genome (11) were found to be the effector protein substrates of the Icm/Dot system (12). Phylogenetic analyses suggest that these genes would have been acquired by interkingdom horizontal gene transfer (HGT) during coevolution of *Legionella* and its natural host for millions of years (13). Hundreds of genome sequences of L. pneumophila clinical isolates have now revealed that recombination events are common in this species (14–16). Thus, intraspecies and interkingdom HGT events are playing a major role in the evolution and adaptation of this species.

The high degree of plasticity of the genomes of L. pneumophila is consistent with the fact that it is competent for natural transformation (17). Natural transformation refers to the ability of certain bacteria to capture exogenous DNA and integrate it into their genomes by homologous recombination (18). It is one of the driving forces for bacterial evolution that can lead to the emergence of new pathogenic bacteria and new antibiotic-resistant recombinants. It is a widespread mechanism of HGT in bacteria, with more than 80 experimentally confirmed transformable species (19). The DNA uptake mechanisms and associated proteins constituting the so-called DNA uptake machinery are highly conserved (18), suggesting that most species are potentially transformable. DNA uptake first involves a type IV pilus (T4P) (20) whose direct observation supports a model in which it binds DNA via its tip and its retraction allows the internalization of DNA into the periplasm (21). The periplasmic DNA-binding protein ComEA serves as a ratchet (22, 23), and large amounts of DNA can accumulate in the periplasm before being converted into single-stranded DNA (ssDNA) and translocated across the cytoplasmic membrane through the ComEC inner membrane channel (24). In the cytoplasm, the ssDNA is protected by the transformation-dedicated protein DprA (25) and the single-stranded binding protein SsbB (26). If the internalized ssDNA possesses regions homologous to those in the bacterial chromosome, it is integrated by homologous recombination mediated by the recombinase RecA, which interacts with DprA (27). In Gram-negative bacteria, the newly discovered ComM helicase is also involved in this recombination process (28).

In most transformable species, these proteins are not expressed constitutively but only when the bacterium is in a genetically programmed and transient state called competence (29). L. pneumophila was first reported to be competent when grown at 37°C under some form of stress, such as under microaerophilic conditions (17) or with exposure to DNA-damaging agents (30). In the absence of any stress, L. pneumophila becomes transiently competent when grown at 30°C at the transition between the exponential and stationary growth phases (31, 32). L. pneumophila is unique in that the regulation of competence does not involve transcriptional activation of the competence regulon. Rather, the core genes encoding the DNA uptake system (*comEC*, *comEA*, *comFC*, and *comM*) are subjected to posttranscriptional repression by a ribonucleoprotein complex consisting of a small RNA, RocR, and an RNA chaperone, RocC (32). At the onset of the stationary phase, the expression of RocR decreases and the translation of the mRNAs encoding the DNA uptake system allows L. pneumophila to take up and recombine extracellular DNA. Most L. pneumophila clinical isolates do transform under these conditions, yet some isolates fail to develop competence, and in some instances, this is due to the presence of a mobile genetic element (MGE) that encodes a RocR homolog that acts as a substitute of the chromosome-encoded RocR (33). Competence is further repressed in stationary phase by the quorum-sensing system (34, 35). The regulation of competence in L. pneumophila remains poorly understood (36).

Regulation of competence is best understood in the Gram-positive Streptococcus pneumoniae, in which the comprehensive genetic approach of transposon insertion sequencing (TIS) has recapitulated decades of findings (37). Beyond the identification of additional regulatory or functional elements of natural transformation, this approach gave rise to a better understanding of the biology of this bacterium by identifying genes involved in virulence and in resistance against stresses. TIS approaches encompass a number of similar methods (transposon sequencing [Tn-seq], transposon-directed insertion site [TraDIS] sequencing, insertion sequencing [INseq], and high-throughput insertion tracking by deep sequencing [HITS]) (38–41) that have been used for the identification of essential genes on a genome-wide scale in a number of species (42). TIS relies on the mapping and quantification of transposon insertion mutants by high-throughput DNA sequencing, and a critical factor is to obtain high-saturation libraries of transposition mutants (43). TIS was recently applied to L. pneumophila with a focus on effector-encoding genes and their conditional involvement in intracellular replication (44, 45). However, the libraries of mutants were either targeted for effectors (44) or of low coverage (45). Thus, the full power of TIS has not yet been harnessed to understand fundamental or specific aspects of the biology of L. pneumophila, possibly because of the difficulty of obtaining high-saturation mutant libraries. In addition, the current libraries were constructed in strain lp02, which has lost competence regulation during its laboratory domestication (46). Here, we sought to obtain a high-coverage library for Tn-seq in L. pneumophila that could be used to apprehend the genetic basis of the many life traits of this species. We found that some clinical isolates of L. pneumophila are more permissive to transposon mutagenesis than the commonly used laboratory strains. We obtained a high-coverage Tn-seq library in an unaltered clinical isolate and identified genes essential for fitness and growth in axenic medium. We then applied Tn-seq to identify the genes involved in competence and natural transformation.

## RESULTS AND DISCUSSION

### High-saturation Tn-seq library of L. pneumophila.

With the objective of obtaining a Tn-seq library of L. pneumophila, we tested the conjugative delivery of the Himar1-based transposon carried by the *pir*-dependent mobilizable plasmid pBT20 to the commonly used strain Paris. Conjugation assays with the MFDpir donor strain produced only a few insertional mutants. We hypothesized that the Paris strain was particularly resistant and tested 12 other clinical isolates belonging to sequence type 1 (ST1). Similarly to the Paris strain, none of the ST1 isolates generated a meaningful number of mutants. We concluded that for an unknown reason, the ST1 isolates (which would include the Philadelphia-1 derived laboratory strains lp02 and JR32) were poorly permissive to conjugative transfer and/or to transposition by Himar1. We thus tested 8 other non-ST1 clinical isolates. We obtained several thousand mutants for 5 of these. We decided to continue with isolate HL-0709-3014, for which we obtained a complete genome composed of a circular chromosome of 3,405 kb and a plasmid of 106 kb (see Materials and Methods). A total of 3,183 open reading frames were detected, 2,791 and 2,741 of which have orthologs in the Paris and Philadelphia-1 strains, respectively (see Data Set S1 in the supplemental material). HL-0709-3014 belongs to the ST18 lineage, which is closely related to the ST1 lineage. Hence, it is phenotypically similar to the Paris strain, it is naturally transformable, and it shows similar intracellular replication rates in amoebae (Fig. S1). It also effectively replicates in human and murine macrophages (>2-log growth in 72 h) (Fig. S1). We isolated HL77S, a spontaneous streptomycin-resistant mutant of HL-0709-3014, and subjected it to mutagenesis with the transposon of pBT20. This mariner-based transposon inserts at TA sites and includes an outward facing P*tac* promoter that can minimize possible polar effects on operon and downstream genes. About 250,000 colonies of mutants were isolated on charcoal-yeast extract (CYE) plates and collected (initial isolation). The library was then cultured in rich medium at 30°C and reisolated on CYE (second isolation). Sequencing of the transposon insertion sites revealed a maximum of 110,679 unique insertion out of 255,021 possible TA sites (43% saturation) and an average of one insertion site every 31 bp. This represents a significant improvement over the previously published library in the ST1 strain lp02, which contained 17,781 unique insertion sites (7% saturation) (45). Thus, we obtained a high-saturation Tn-seq library in an L. pneumophila clinical isolate that can be used as a surrogate for the commonly used laboratory strains (Paris, JR32, lp02, and AA100).

### Analysis of gene essentiality.

**FIG 1:**
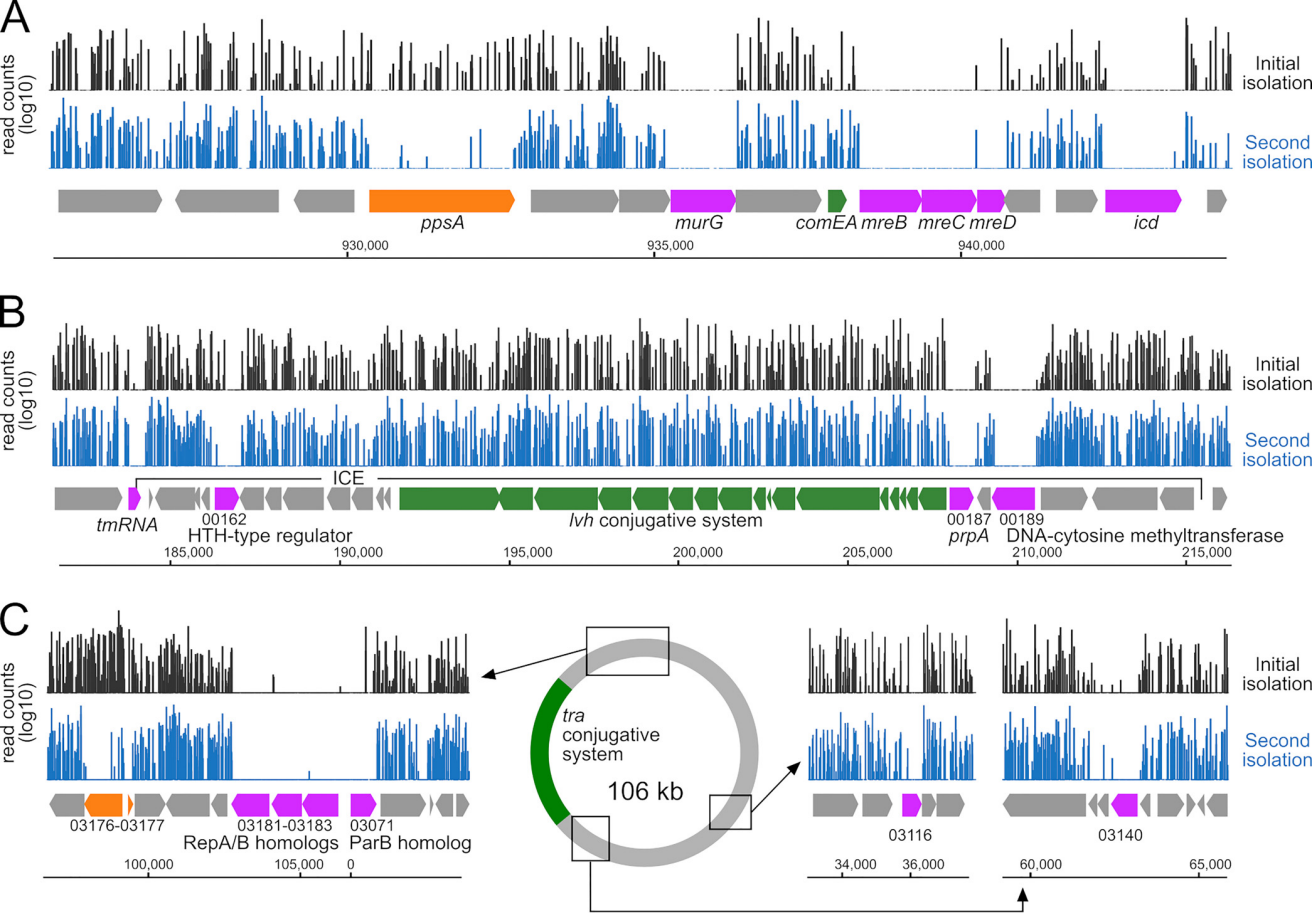
Tn-seq analysis of L. pneumophila strain HL77S. (A) Log_10_ read counts of transposon insertions after initial library isolation (black) and second isolation (blue). Genes identified as essential are in magenta, and fitness determinants are in orange. Other genes of interest are in green. (B) Transposon insertion coverage in a region encompassing an integrative conjugative element (ICE) harboring essential genes (magenta) and genes encoding a conjugative system (green). The duplicated sequence GCGGGTTCGATTCCCGCCGCCTCCACCA of the tmRNA and located 66 kb away delineates the boundaries of the ICE. (C) Essential genes and fitness determinants in the conjugative plasmid of HL77S.

The high saturation allowed the identification of genes essential for growth. To do so, we used two statistical methods: the Gumbel method (47), a Bayesian model based on longest consecutive sequence of TA sites without insertion in the genes, and the hidden Markov model (HMM) method based on the detection of genes with unusually low read counts (48). Both methods gave similar results with 401 (Gumbel) and 500 (HMM) genes identified as essential, 382 of which were identified as strictly essential by both methods (Data Set S1), consistent with the average number (391) of essential genes identified by TIS in other bacterial species (49). This is lower than the number of essential genes (588) identified by the TraSH method in the lp02 strain of L. pneumophila, but these also included fitness determinants (50). The data confirmed our previous observation of the essentiality of the actin-like protein MreB (51) but also that of MreC and MreD, while intergenic insertions between *mreC* and *mreD* are tolerated (Fig. 1A).

Comparative analysis of the second and initial isolation identified 181 genes which were nonessential at the initial isolation but whose inactivation impaired fitness (log_2_ fold change [log_2_FC] < −2; *P* < 0.05). These include the gene encoding exoribonuclease R, whose growth defect was previously reported (52), and the RNA chaperone Hfq but also, more surprisingly, the substrates of the Icm/Dot type IV secretion AnkQ and SdbB (Data Set S1). Presumably because their inactivation lowered the fitness so dramatically, 61 genes not essential after the initial isolation were deemed essential on the second isolation. These include the phosphoenolpyruvate synthase-encoding gene *ppsa* (Fig. 1A) and the genes encoding the sigma factor RpoS, the recombinase RecA, the tyrosine recombinase XerC (which is involved in chromosome dimer resolution), and the transfer-messenger RNA (tmRNA)-binding protein SmpB, which is involved in *trans* translation. Indeed, this is consistent with our previous demonstration that *trans* translation is essential in L. pneumophila (53) and that no insertions are observed in the tmRNA-encoding gene (Fig. 1B).

The vast majority of the essential genes that encode proteins have orthologs in the Paris and Philadelphia-1 genome, as expected for genes that encode proteins involved in the fundamental processes of the cell. However, of all the genes found to be essential on either the initial or second isolation, 14 have no orthologs in the Paris and Philadelphia-1 strains. How could strain-specific genes be essential? Three of these genes (HL77S_01135, HL77S_01141, and HL77S_01146) encode the antitoxin component of toxin-antitoxin (TA) modules clustered within a 4-kb segment. HL77S_01068 is located next to a gene encoding a toxin of the type II TA system, suggesting that it also encodes an antitoxin. Others have no known function, such as HL77S_02141, which encodes a protein with a predicted helix-turn-helix (HTH) motif, or HL77S_00079, encoding a protein with a conserved domain of unknown function (DUF3800).

Consistent with their being part of the accessory genome, genomic comparison with other complete genomes of L. pneumophila indicates that all of these genes reside in highly variable regions, often in proximity to putative transposase and prophage integrase. However, no genetic structure corresponding to a complete mobile genetic element (MGE) could be detected. In contrast, two essential genes (HL77S_00162 and HL77S_00189) are within a recognizable MGE corresponding to an integrative conjugative element (ICE) inserted at the 3′ end of the tmRNA-encoding gene and carrying a conjugative system homologous to the Lvh system (Fig. 1B). HL77S_00162 is an HTH-type regulator and HL77S_00189 is predicted to encode a DNA-cytosine methyltransferase. The ICE shows a third essential gene (HL77S_00187, *prpA*), conserved in the Lvh ICE of the Paris strain and encoding a LexA/CI-like repressor homolog (Fig. 1B). Another strain-specific essential gene (HL77S_00197) is located just downstream of the ICE, in a unique region that may represent a remnant of another MGE. Another three of the 14 strain-specific essential genes (HL77S_03181, HL77S_03182, HL77S_03183) are part of the 106-kb conjugative plasmid and clustered with another essential gene (HL77S_03071) which has a homolog on the pLPP plasmid of the Paris strain (Fig. 1C). Encoding Rep or Par homologs, these genes are involved in replication/partition of the plasmid, and their inactivation likely resulted in plasmid loss.

Also on this plasmid, two other genes of unknown function also appear essential, one that is unique and has no conserved domain (HL77S_03140) and one with a homolog on pLPP (HL77S_03116, *plpp0094*) containing an N-terminal HTH motif and a C-terminal nucleotidyltransferase (NT) domain also found in DNA polymerase beta (Fig. 1C). Insertions in two divergently oriented genes (HL77S_03176 and HL77S_03177) are also associated with strong fitness defects and are of unknown function (Fig. 1C).

Overall, we found that many essential genes can be found within MGEs. Insertion in genes controlling vertical transmission can result in the loss of the MGE (and thus of transposon insertions, making the corresponding gene seemingly essential). This might be the case for repressor of excision of ICE or genes required for replication/partition of plasmids. Other genes might be required to limit the cost of the MGE to the fitness of their host. This might be the case for the LexA/CI-like repressor of ICE, as exemplified by the Vibrio cholerae SXT ICE, for which inactivation of the LexA/CI-like repressor SetR is deleterious to its host (54). Whatever the mechanism, the genes characterized as essential in MGEs ensure their vertical transmission. Thus, our result indicates that in addition to identifying genes required for the fundamental functions of the cell, Tn-seq analyses can also reveal novel genes that contribute to vertical transmission of MGEs, representing an untapped resource to study bacterium-MGE coevolution.

### Tn-seq analysis of natural transformation in L. pneumophila.

We sought to use the Tn-seq library to identify all genes required for competence and subsequent natural transformation. Mutants defective for expression of competence, DNA uptake, protection, or recombination would not be able to undergo transformation and would thus be missing in the transformed population. We subjected the Tn-seq library to natural transformation with two distinct transforming DNAs carrying a kanamycin resistance cassette inserted in the *legK2* gene (encoding an Icm/Dot substrate) or in the *ihfB* gene (encoding the B subunit of the integration host factor [IHF]). This strategy should limit false-positive results arising from epistatic interactions between the gene in which the selected resistance cassette is inserted (*legK2* or *ihfB*) and the transposon-disrupted genes.

**FIG 2:**
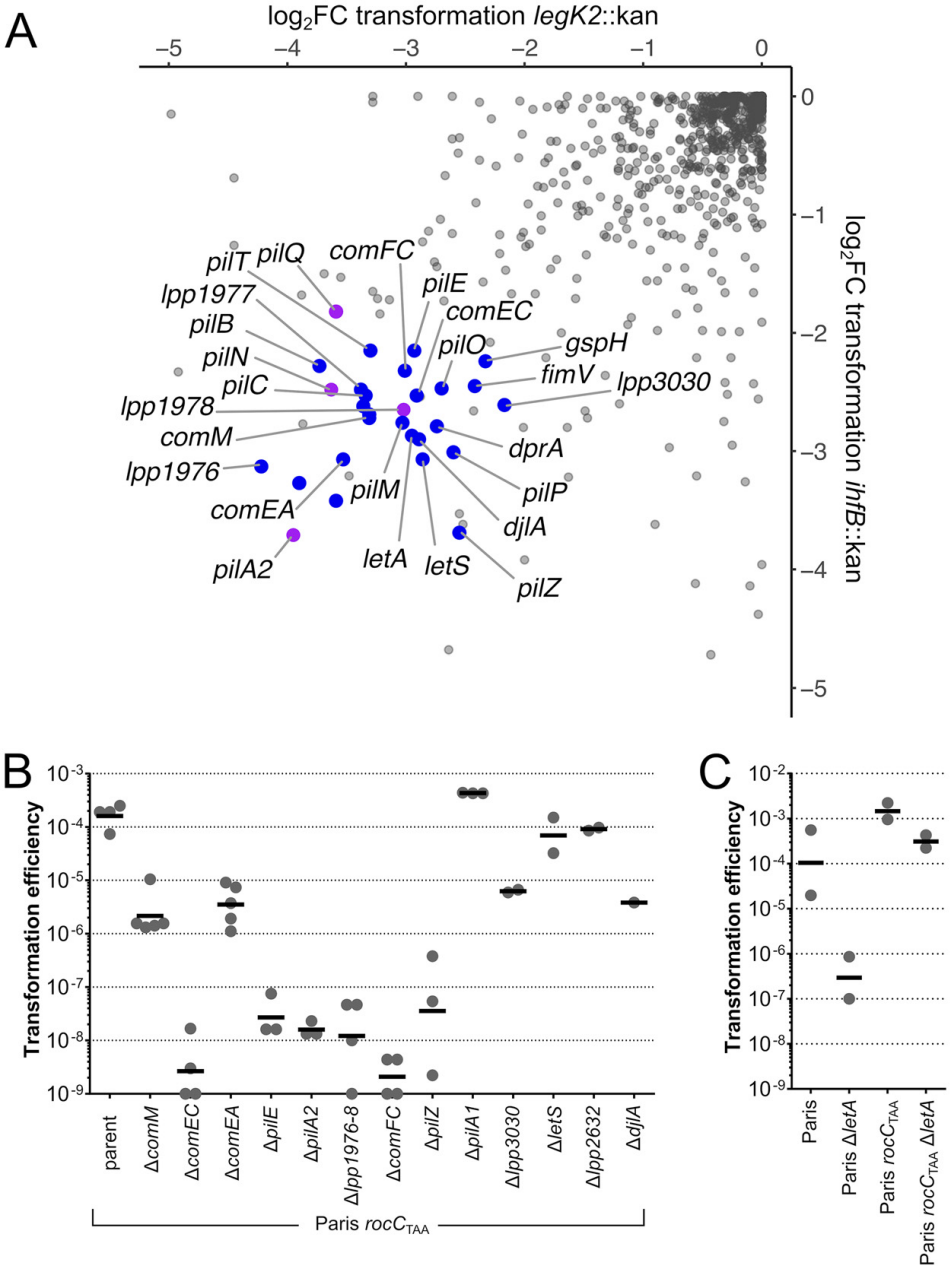
Identification of genes required for natural transformation by Tn-seq. (A) Scatterplot of fold change (log_2_) of insertions in the corresponding genes in two tested transformation conditions. HL77S was subjected to natural transformation with a 4-kb PCR fragment of the *legK2* or *ihfB* genes interrupted by a kanamycin resistance gene. Reads counts per gene were determined and expressed as fold change between the nontransformed population and the *legK2*::*kan*- or *ihfB*::*kan*-transformed populations. Individual genes (gray dots) were considered to be required for natural transformation if log_2_FC was >2 or <−2 and if *P* was <0.05 under one (magenta dots) or both (blue dots) conditions. (B) Natural transformation efficiency of reconstructed mutants in the Paris *rocC*_TAA_ strain, which is constitutively competent for natural transformation. Transformation experiments were performed at least three times independently, and transformation frequencies were plotted (gray dots) along with the geometric means (black lines). (C) Natural transformation efficiency of the reconstructed Δ*letA* mutant in the original Paris strain and the constitutively competent Paris *rocC*_TAA_ strain. Transformation experiments were performed twice independently, and transformation frequencies were plotted (gray dots) along with the geometric means (black lines).

Transformation frequencies of the HL77S Tn-seq library were in the range of 1 × 10^−5^ to 6 × 10^−4^, and to avoid a bottleneck effect (43), we collected over 5 × 10^6^ transformants. The control, nontransformed populations were subsampled to obtain a similar number of isolated colonies. We observed 28 genes in which insertions cause a decrease (log_2_FC < 2; *P* < 0.05) of the mutants in the population transformed with either the *legK2*::Kan or the *ihfB*::Kan DNA (Fig. 2A). As expected, these include the gene encoding the periplasmic DNA receptor ComEA and the genes required for DNA transport across the inner membrane (*comEC* and *comFC*), for ssDNA protection in the cytoplasm (*dprA*), and for recombination (*comM*). Many of the other genes encode factors known to be involved in type IV pilus assembly, confirming the role of this system in natural transformation of L. pneumophila (17). These include the retraction ATPase PilT (lpp1995), the extension ATPase PilB, the PilQ secretin, the PilC platform protein and the proteins of the PilMNOP complex. Interestingly, we observed no transformation defect for insertions in the gene encoding another putative pilus retraction ATPase (lpp2271). Two putative pilins, PilE (lpp0681) and PilA2 (lpp1890), were also identified as essential for transformation.

Other genes potentially involved in natural transformation or regulation of competence were also identified (*lpp1976*, *lpp1977*, *lpp1978*, *lpp3030*, *lpp2632*, *djlA*, *letA*, and *letS*). In order to confirm their role and disentangle their involvement in DNA uptake or in regulation, we constructed gene deletion mutants in a Paris strain with a premature stop codon of the RocC chaperone (Paris *rocC*_TAA_) which is defective for repression of competence and constitutively transformable (32). Deletion mutants corresponding to genes known to be involved in natural transformation were defective for transformation as expected (Fig. 2B). The *comEC* and *comFC* mutants were totally defective for transformation, and the *comM* and *comEA* mutants showed an ∼100-fold decrease in transformation frequencies, as observed for other species (28, 55). Similar partial transformation defects were observed for mutants of *djlA*, encoding a DnaJ-like protein required for intracellular replication in Legionella dumoffii (56), and *lpp3030*, a *Legionellaceae*-specific gene encoding an uncharacterized protein with a putative signal peptide. However, in this constitutively competent background, we could not confirm the involvement of *lpp2632*, which encodes a glutaryl coenzyme A (glutaryl-CoA) dehydrogenase, indicating that this gene is dispensable for the transformation process (Fig. 2B). Mutants of this gene show a reduced fitness (log_2_FC = −1.99; *P* < 0.01) (Data Set S1), suggesting that the transformation defect observed in the Tn-seq analysis is an indirect consequence of the mutants limited growth that could prevent entry into the competence state at the onset of the stationary phase.

Intriguingly, in the constitutively competent strain, a deletion mutant of *letS* also showed no transformation defect (Fig. 2B). LetS is the sensor of the LetA/LetS two-component system (TCS) homologous to the BarA/UvrY system in Escherichia coli (57) and GacS/GacA in *Pseudomonas* spp (58). In L. pneumophila, the LetA/LetS system has been identified for the first time in a screen of mutants deficient in the expression of flagellin (59) and has since been shown to be involved in the activation of various virulence traits as well as intracellular growth in amoebae (60–63). One of the major roles of the LetA/S TCS is to enable the transition from the transmissive to the replicative phase (64). The fact that both LetA and LetS are output together in the transformation screens while the *rocC*_TAA_ Δ*letS* mutant is not defective for transformation suggests that this TCS is involved in the regulation of competence in L. pneumophila. To test this, we reconstructed insertion mutants of the *letA* gene, encoding the response regulator/activator of this TCS, in the Paris strain and *rocC*_TAA_ genetic backgrounds and tested them for their ability to undergo transformation. Consistent with the Tn-seq data, inactivation of LetA in the Paris strain reduced transformability by over 500-fold (Fig. 2C). In contrast, like the Δ*letS* mutant, the Δ*letA* mutant in the constitutively competent strain *rocC*_TAA_ background is only marginally affected for natural transformation (Fig. 2C). These data suggest that the LetA/S TCS is involved in the regulation of competence upstream of the regulation controlled by the RocC/RocR system. Further work will be needed to determine the precise role of this TCS and the associated regulatory cascades in the regulation of L. pneumophila competence.

### Major and minor pilins required for natural transformation.

With the remarkable exception of Helicobacter pylori (65), in all Gram-negative bacteria DNA uptake requires type IV pili (20). Type IV pili are extracellular filaments resulting from the assembly of thousands copies of an abundant major pilin but also of less abundant minor pilins that could be embedded in the filaments (core minor pilins) or at its tip (noncore minor pilins) (66, 67). The nomenclature of pilins is relatively confusing, but the major pilin is generally called PilA, although in *Neisseria* spp., that protein is called PilE (67). In addition, some species carry multiple copies of pilins, and at least in Thermus thermophilus, two major pilins (PilA4 and PilA5) are assembled into distinct filaments, required for natural transformation and twitching motility, respectively (68). The L. pneumophila genomes show two putative PilA homologs encoded by two consecutive genes (*pilA1* [*lpp1889*] and *pilA2* [*lpp1890*]) in a locus removed from any other genes encoding type IV pilus components. Both the Tn-seq data and reconstructed mutants show that PilA2 is required for natural transformation, while PilA1 is dispensable (Fig. 2A and B). The two copies of PilA-encoding genes may have resulted from a gene duplication event, followed by the loss of function of one of the two copies.

**FIG 3:**
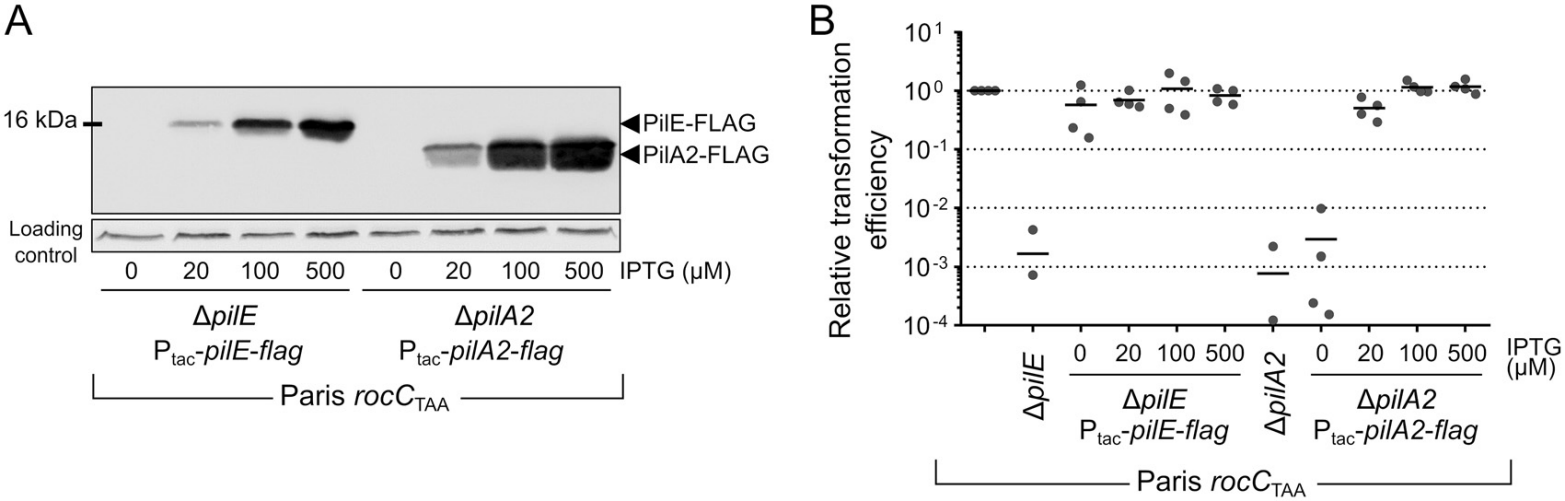
PilA2 is the major pilin of the L. pneumophila transformation pilus. (A) Western blot analysis of ectopically expressed PilA2-FLAG (encoded by p1890F) and PilE (encoded by p0681F) as a function of the IPTG inducer. (B) Complementation of the Δ*pilE* and Δ*pilA2* mutants in the Paris *rocC*_TAA_ strain by the ectopic expression of PilA2-FLAG (encoded by p1890F) and PilE (encoded by p0681F). Transformation frequencies were determined four times independently as a function of the IPTG inducer and normalized to 1 for the parental strain (Paris *rocC*_TAA_).

The Tn-seq data show that a putative pilin PilE (Lpp0681) appears to be required for transformation, while five genes upstream of *pilE* (*lpp0686* to *lpp0682*) and respectively annotated as encoding PilC/PilY1 and minor pilins PilX, PilW, PilV, and GspH/FimT appear to be dispensable. Targeted gene deletion also confirmed the Tn-seq result that PilE is required for natural transformation, corroborating an initial observation that a mutant of the *pilE* gene (then designated *pilE_L_*) is not competent for transformation (17). Based on sequence comparison with PilA from Pseudomonas aeruginosa, *pilE_L_* was then proposed to encode a type IV pilin structural gene (69). We thus investigated which of PilE and PilA2 constitute the major pilin in L. pneumophila. We tested the complementation of the Δ*pilE* and Δ*pilA2* deletion mutants obtained in the constitutively competent *rocC*_TAA_ strain. Both *pilE* (*lpp0681*) and *pilA2* (*lpp1890*) were ectopically expressed from an IPTG (isopropyl-β-d-thiogalactopyranoside)-inducible promoter to produce fusion proteins with a C-terminal FLAG epitope. Western blot analysis showed that both PilE-FLAG and PilA2-FLAG could be expressed in an IPTG-dependent manner, with PilE-FLAG always being expressed at a higher level than PilA2-FLAG, likely reflecting the efficiency of their ribosome-binding sites (Fig. 3A). Data show that a low expression of PilE-FLAG is sufficient to restore natural transformation in the *rocC*_TAA_ Δ*pilE* mutant, as full complementation of the transformation phenotype is obtained even in the absence of IPTG (Fig. 3B). In contrast, a higher concentration of IPTG and thus a higher expression of PilA2-FLAG are required to obtain a functional complementation of the *rocC*_TAA_ Δ*pilA2* mutant (Fig. 3B).

**FIG 4:**
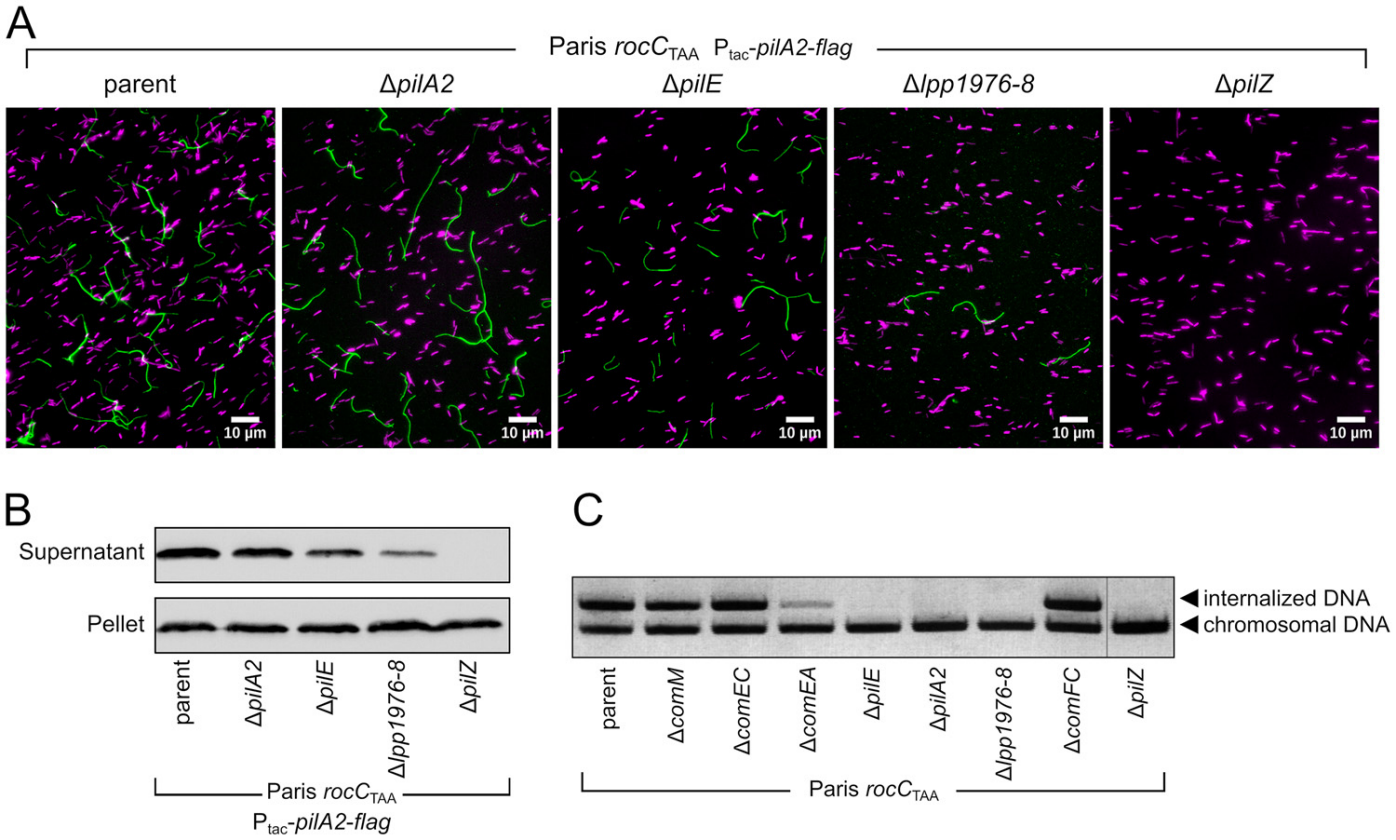
PilA2 assembly into extracellular filaments depends on *pilE*, the operon *lpp1976–lpp1978*, and *pilZ*. (A) Visualization of PilA2-FLAG filaments (green) by immunofluorescence microscopy using fluorescein-conjugated anti-FLAG antibody. Bacteria were visualized by labeling DNA with Hoechst 33288 (magenta). (B) Western blot detection of extracellular PilA2. Bacteria were vortexed to release pili which were precipitated from supernatants. PilA2-FLAG was detected in supernatant and whole-cell lysates (pellet) using anti-FLAG antibodies. (C) DNA uptake assay of the reconstructed mutants defective for natural transformation. The transformation-deficient mutants were tested for the ability to internalize pGEM-HYG1, a nonreplicative plasmid. Following incubation with the DNA and subsequent DNase I treatment, the internalized DNA was detected in cells by PCR for pGEM-HYG. As a control, chromosomal DNA was also detected by PCR. This multiplex PCR was analyzed by agarose gel electrophoresis and labeling of DNA with ethidium bromide. Images are representative of experiments performed three times independently.

The results are consistent with a model in which PilA2 is the major pilin while PilE is a low-abundance minor pilin. In addition, when expressed ectopically in the *rocC*_TAA_ strain, PilA2-FLAG assembles in long extracellular filaments (Fig. 4A and B). In the *rocC*_TAA_ Δ*pilE* strain, fewer PilA2-FLAG filaments are observed by microscopy, and Western blotting confirmed a lower abundance of extracellular PilA2-FLAG (Fig. 4A and B). This indicates that PilE, while not strictly essential, still plays a role in pilus formation. Minor pilins have been proposed to localize at the tip of the pilus and stabilize it (66). In Vibrio cholerae, DNA binding has been observed to occur at the tip of the pilus (21). Because PilE is not strictly essential for pilus assembly but required for transformation and DNA internalization (Fig. 3B and 4C), we propose that PilE is the DNA receptor at the tip of a pilus composed of PilA2 subunits.

### Genes of unknown function and *pilZ*.

In addition to the pilin mutants that were strongly defective for natural transformation, we investigated the underlying reason for the strong transformation defect of the mutant with a deletion of the operon *lpp1976*-*lpp1977*-*lpp1978* (Fig. 2B). Consistent with being important for natural transformation, this operon was found to be upregulated in the constitutively transformable *rocC*_TAA_ mutant (32). Automated annotation did not assign a predicted function for the three genes, and a BLAST search failed to identify homologs outside the genus *Legionella*. The mutant with the deletion of the entire operon (Δ*lpp1976*–*lpp1978*) was unable to take up DNA (Fig. 4C), indicative of a defect in type IV pilus-mediated DNA import. Indeed, in this mutant, the levels of extracellular PilA2-FLAG were strongly reduced (Fig. 4B). The mutant produced few, and short, PilA2-FLAG filaments (Fig. 4A), revealing a major defect in type IV pilus assembly or stability. A search for conserved domains in the three predicted proteins identified, in the 268-amino-acid Lpp1977, only a partial homology with the N-terminal part of the Tfp pilus assembly protein PilW. This suggested that *lpp1976*-*lpp1977*-*lpp1978* would encode a set of minor pilins. Indeed, PilFind (70) identified an N-terminal transmembrane segment in all three predicted proteins and a type II signal in Lpp1977 and Lpp1978. The operonic organization of these three genes is reminiscent of the operon encoding four minor pilins of the type IV pilus of Neisseria meningitidis (*pilHIJK*) and P. aeruginosa (*fimU-pilVWX*) and of the type II secretion system (T2SS) of enterotoxigenic E. coli (*gspHIJK*). In the latter system, the last three genes (*gspIJK*) encode minor pseudopilins which assemble into a stable complex (71). This complex of minor pilins would form in the inner membrane to establish a platform for the assembly of the major pilin (72) and remain at the tip of the pilus, stabilizing it (66). Such heterotrimeric complex may be formed by minor pilins of limited homology but displaying structural similarity (73). Altogether, this supports the hypothesis that in *Legionella* species, the initiation complex of the transformation pilus is formed by Lpp1976, Lpp1977, and Lpp1978, which serve as a scaffold for assembly of the major pilin PilA2.

Another gene whose deletion resulted in strong deficiency in natural transformation is *pilZ*. The Δ*pilZ* mutant is defective for DNA uptake and is totally unable to produce extracellular PilA2 or assemble PilA2 filaments (Fig. 4). PilZ was originally identified in P. aeruginosa as required for the secretion of PilA polymers, pilus genesis, and type IV pilus-dependent motility (74). Although the P. aeruginosa PilZ served as the founding member of a diverse family of proteins with PilZ domains (75), some of which bind the cyclic di-GMP (c-di-GMP) second messenger, it itself does not bind c-di-GMP (76). A *pilZ* mutant in Xanthomonas campestris pv. campestris displays a minor defect in type IV pilus-dependent motility (77), and this PilZ ortholog directly binds to the PilB ATPase and the c-di-GMP-interacting FimX protein (78). However, no homolog of FimX could be identified in L. pneumophila, and Tn-seq did not reveal any c-di-GMP synthesis enzyme required for natural transformation. However, Tn-seq did show that PilB was important for transformation (Fig. 2A). We thus speculate that, in L. pneumophila, PilZ controls type IV pilus assembly independently of c-di-GMP signaling and through a direct interaction with PilB.

### Conclusion.

We report on a clinical isolate of L. pneumophila, which displays phenotypes (intracellular replication and competence for natural transformation) similar to those of commonly used laboratory strains. In contrast to laboratory strains, a high-saturation Tn-seq library could be obtained and allowed to define essential genes, including strain-specific genes in MGEs. Tn-seq analyses of transformation, with follow-up work performed in the Paris strain, defined the set of major and minor type IV pilins that are engaged in DNA uptake. While we focused here on mutants that were strongly deficient for natural transformation, Tn-seq also identified potential regulators of competence as well as genes of unknown function that also participate in natural transformation (for instance, *djlA* and *lpp3030*). We demonstrate here that strain HL77S could represent a surrogate for the commonly used lab strains to perform Tn-seq analysis. Unleashing the full power of Tn-seq is a major step toward the identification of the genetic basis of traits that turned L. pneumophila into a successful pathogen, such as its ability to form biofilms and to resist biocides and unicellular predators.

## MATERIALS AND METHODS

### Bacterial strains and growth conditions.

Legionella pneumophila strains were grown in liquid medium containing ACES [*N*-(2-acetamido)-2-aminoethanesulfonic acid] and buffered yeast extract (AYE) or on solid medium plates containing ACES and buffered charcoal yeast extract (CYE). When appropriate, kanamycin, gentamicin, and streptomycin were added at 15 μg/ml, 10 μg/ml, and 50 μg/ml, respectively. Clinical isolates of L. pneumophila, including HL-0709-3014 (referred to as HL77), were provided by the Centre National de Référence des Légionelles, Lyon, France. A streptomycin-resistant of HL-0709-3014 mutant was obtained by plating 1 ml of culture on a streptomycin-containing CYE plate and was named HL77S. Escherichia coli MFDpir (79) with a chromosome-integrated RP4 conjugative system was used as a donor strain for conjugative transfer of the mutagenesis system pBT20 (80), which carries a Himar1 transposon bearing a gentamicin resistance gene and an outward-facing promoter. MFDpir is auxotrophic for diaminopimelic acid (DAP) and thus was always cultivated with 1% DAP. Axenic Acanthamoeba castellanii cells were grown in proteose-yeast extract-glucose (PYG) medium at 30°C and split once or twice a week. Human U937 cells were maintained in RPMI 1640 with 10% heat-inactivated fetal calf serum and 1% penicillin-streptomycin at 37°C and 5% CO_2_. Differentiation into macrophages was induced by the addition of PMA (phorbol 12-myristate 13-acetate) at a final concentration of 100 ng/ml. Murine macrophages, RAW 264.7, were cultured in Dulbecco’s modified Eagle medium (DMEM) supplemented with 10% fetal bovine serum (FBS) and 1% penicillin-streptomycin at 37°C and 5% CO_2_.

### Intracellular growth experiments.

The ability of HL77S strain to infect host cells was compared to that of the Paris strain. Paris Δ*dotA* was used as a negative control, as this mutant is unable to multiply in host cells. The ability of HL77S to replicate in the amoeba Acanthamoeba castellanii was determined as follows. Amoebas were resuspended in PYG medium at a concentration of 1 × 10^6^ amoebas/ml. The suspension was distributed in a flat-bottom 6-well plate (2 ml per well; 2 × 10^6^ amoebas per well) and incubated for 3 h at 30°C to allow amoebas to settle and adhere to the plate. One milliliter of a PY medium suspension containing 2 × 10^6^ bacteria (from a culture in stationary phase, optical density [OD] of ∼5) were added in each well to obtain a multiplicity of infection (MOI) of 1. The plate was centrifuged for 10 min at 650 × *g* and incubated at 30°C for 72 h. At 0 h, 48 h, and 72 h, 250 μl of supernatant of each well was serially diluted, spotted onto CYE plates, and incubated at 37°C for 72 h to determine the number of CFU per milliliter.

The ability of L. pneumophila strains to infect macrophages was determined as follows. Overnight cultures of bacterial strains (optical density [OD] of ∼5 in AYE medium) were diluted (1:10) in the appropriate cell culture medium (DMEM for RAW 264.7 cells and RPMI 1640 for U937 cells) and incubated for 1 h at 37°C. Host cells (differentiated U937 and RAW 264.7 cells) were seeded in 24-well plates, 3 wells per condition. Cells were washed and were infected with bacteria at an MOI of 1 or 10. Plates were centrifuged at 500 × *g* for 5 min to promote bacterium-cell contact and incubated at 37°C and 5% CO_2_ for 24 h to 72 h. Every 24 h, the contents of one well per condition was transferred to a 1.5-ml tube and centrifuged at 16,000 × *g* for 5 min. The pellet containing the infected macrophages was resuspended in sterile distilled water to lyse the macrophages and release the bacteria. The suspension was serially diluted, spotted onto CYE plates, and incubated at 37°C for 72 h to determine the titer, in CFU per milliliter.

### Plasmid and strain constructions.

Plasmid pJET1.2-legk2::kan, used for natural transformation experiments, was constructed by cloning a 6-kb fragment consisting of the *legK2*::*kan* gene (81) and 2 kb of its flanking regions in the pJET1.2/blunt cloning vector (Thermo Fisher) according to the manufacturer’s instructions. All the mutants generated in this study are derived from L. pneumophila Paris or L. pneumophila Paris *rocC*_TAA_. All the genes suspected to be involved in natural transformation were deleted by replacement with a kanamycin resistance gene. To do so, the upstream (PCR product A [PCRA]; 2 kb) and downstream (PCRC; 2 kb) regions of each suspected gene were amplified, respectively, with the primer pairs X_P1/X_P2-tail-pKD4 and X_P3-tail-pKD4/X_P4 (where X designates the gene to be deleted). X_P2-tail-pKD4 and X_P3-tail-pKD4 carry 30-nucleotide sequences complementary to the ends of the kanamycin cassette. This complementarity was used to assemble PCRA and PCRC to the kanamycin resistance cassette (PCRB; 1,490 kb amplified from plasmid pGEMPKD4 (32) with primer pair pKD4_P1/pKD4_P2) by overlap extension PCR. Overlapping PCR products were naturally transformed in the desired strain. Transformants were selected on CYE supplemented with kanamycin (15 μg/ml). Integration of the *kan* cassette at the correct locus was finally verified by colony PCR. Plasmids p1890F and p0681F, encoding the FLAG-tagged PilA2 and PilE, were constructed by amplifying *lpp1890* (*pilA2*) and *lpp0681* (*pilE*) with primers lpp1890-F/lpp1890F-R and lpp0681-F/lpp0681F-R, respectively. The PCR products and the recipient plasmid pMMB207C were digested with HindIII/BamHI and ligated to place the genes under the control of the P*tac* promoter. All strains, plasmids, and oligonucleotides are listed in Table S1 in the supplemental material.

### Generation of a transposon insertion mutant library of Legionella pneumophila.

Transposon-based random mutagenesis was performed as previously described (82) by conjugative delivery of the Himar1-based transposon suicide vector pBT20 from the donor strain E. coli MFDpir to the recipient strain of L. pneumophila to be mutagenized. To do so, both bacteria were cultivated overnight at 37°C with shaking in their corresponding liquid media: 7.5 ml LB broth containing 100 μg/ml ampicillin and 1% DAP for E. coli and 15 ml standard AYE medium for L. pneumophila. Once in stationary phase (OD of ∼5), the L. pneumophila and E. coli cultures were concentrated by centrifugation (5,000 × *g*, 10 min), and cell pellets were resuspended, respectively, in 1.5 ml sterile water and 0.750 ml sterile phosphate-buffered saline (PBS). To promote cell-to-cell contacts and the subsequent conjugation, both concentrated cultures were mixed together by pipetting and spotted on CYE plates without iron or cysteine but supplemented with DAP (CYED) (82) until the sample was exhausted. Plates were incubated at 37°C for 5 to 6 h. All the spots were resuspended in sterile water and used to inoculate transconjugant-selective plates (i.e., CYE plates supplemented with 10 μg/ml of gentamicin). In parallel, the suspension was 10-fold diluted and spotted onto transconjugant-selective plates to evaluate the number of mutants in the library. After 72 h of incubation at 37°C, a mutant library was obtained by collecting all colonies from the plates and resuspending them in AYE–5% glycerol. The suspension was aliquoted and stored at −80°C until its use for a Tn-seq screen. This library, called initial isolation, is named sample XRCR13.

### Natural transformation Tn-seq screen.

Transposon mutants of L. pneumophila HL77S were screened for their ability to undergo transformation. To avoid a bottleneck effect, a volume of the −80°C frozen library containing 10 times the number of mutants in the library was spotted on a CYE plate supplemented with gentamicin (10 μg/ml) and streptomycin (50 μg/ml) to obtain exponentially growing cells. After 24 h of incubation at 37°C, fresh bacteria from the spot were resuspended in AYE to an OD of ∼0.2. This suspension was used to perform transformation assays using 2 μg/ml of either pGEM-ihfB-kan or pGET1.2-legK2-kan as transforming DNA, both conferring resistance to kanamycin. For both transforming DNAs, the transformation screen was conducted in duplicate. The suspensions were cultivated at 30°C with shaking for 40 h to ensure that bacteria underwent transformation and achieved an OD of ∼5. These conditions were expected to give about ∼10^5^ transformants/ml for the DNA conditions. Regarding the no-DNA condition, cultures were diluted in AYE to obtain the same number of CFU on nonselective plates as the output condition on selective plates. Each sample (with DNA and without DNA) was used to inoculate, respectively, nonselective (i.e., CYE) and transformant-selective (i.e., CYE supplemented with 20 μg/ml of kanamycin) plates. In parallel, no-DNA samples were 10-fold diluted and spotted on nonselective plates to determine transformation frequencies as mentioned above. After 72 h of incubation at 37°C, colonies were collected from the plates and resuspended in AYE–15% glycerol until the preparation of DNA libraries. The no-DNA condition is also referred to as the second isolation used in the fitness analysis.

### DNA library preparation and sequencing.

Libraries were prepared as previously described (82). Mutant libraries from the −80°C frozen stock were thawed and centrifuged at maximum speed to pellet them. Genomic DNA (gDNA) extraction was carried out directly on the pellet cells with the Wizard Genomic DNA purification kit (Promega) according to the manufacturer’s instructions. Approximately 30 μg of DNA was mechanically sheared by sonication using a Branson sonifier for 4 min (1 s on and 11 s off; 20% intensity) in 0.5-ml PCR tubes kept on ice. Small gDNA molecules were removed by mixing sonicated gDNA with 0.6× Agencourt Ampure XL magnetic beads (Beckman Coulter) according to the manufacturer’s instructions. These treatments led to gDNA fragments being between 300 and 1,000 bp. Homopolymeric cytosine tails (C-tails) were then added to the 3′ ends of all fragments by incubation of 3 μg of size-selected DNA fragments with the recombinant terminal deoxynucleotidyl transferase (rTdT; 30 U/μl; Promega) at 37°C for 1 h, followed by heat inactivation at 75°C for 20 min. TdT reagents were then removed by purifying the TdT reaction mixture with 1× of Ampure XL beads.

To amplify transposon junctions, a first-round of PCR (PCR1) was performed in a final volume of 50 μl by mixing 500 ng C-tailed DNA, 1 μl biotinylated pBT20-PCR1 primer (30 μM), 3 μl olj376 primer (30 μM), 2.5 μl deoxynucleoside triphosphates (dNTPs) (10 mM), 10 μl Q5 reaction buffer, and 0.75 μl Q5 high-fidelity DNA polymerase (New England Biolabs). PCR1 products were purified using 1× Ampure beads. Biotinylated and purified PCR1 products were then selectively captured using streptavidin-containing Dynabeads (M-280; Invitrogen) according to the manufacturer’s instructions. A second round of PCR was carried out in a final volume of 50 μl by resuspending Dynabeads (which had the PCR1 products bound to them) in the preprepared PCR2 reaction mix consisting of 1 μl pBT20-PCR2 primer (30 μM), 1 μl TdT_index_X primer (30 μM), 2.5 μl dNTPs (10 mM), 10 μl Q5 reaction buffer, and 0.75 μl Q5 high-fidelity DNA polymerase. PCR2 products were purified with 1× Ampure XL beads. The obtained libraries were sequenced on an Illumina HiSeq 4000 in single-end 50 bp using the custom sequencing primer Read1TnLp. Samples and conditions are listed in Data Set S1. Essentiality analysis was performed using reads from sample XRCR13. Fitness analysis was performed by comparing reads from samples XRCR24, -26, -36, and -38 (no-DNA conditions from the transformation screen) versus XRCR13. Analysis of transformation was performed by comparing samples XRCR27 and -39 (*legK2*::*kan* transforming DNA) to samples XRCR26 and -38 (no-DNA control) and by comparing samples XRCR25 and -37 (*ihfB*::*kan* transforming DNA) to samples XRCR24 and -36 (no-DNA control).

### Tn-seq data analysis.

For each condition, 10 to 50 million reads were obtained and trimmed with tools from the Galaxy Project’s public server. Fastx_clipper was used to cut poly(C) tails and remove short reads [<15 bp after poly(C) clipping]. Then, reads were filtered by quality using Trimmomatic and quality checked with FastQC. Trimmed reads in the fastq output file were mapped to the reference genome using the Tn-seq software TPP (Tn-seq preprocessor) (83). Output wig files from TPP were used to perform essentiality analysis using Transit (84). Single-condition essentiality analysis was performed with the HMM (48) or Gumbel (47) method. Conditional essentiality analysis was performed with the “resampling” method according to the Transit software documentation. The complete genome sequence of HL-0709-3014 was obtained (see “Genome sequencing” below) and annotated with Prokka (85). An orthology search was carried out between the strains of L. pneumophila HL77S, Paris, and Philadelphia-1 using the orthology detection eggNOG mapper (86), and COG and KEGG numbers were assigned to each gene.

### Transformation assays.

Natural transformation assays were conducted differently depending on the genetic background of the L. pneumophila strain used.

For the constitutively transformable *rocC*_TAA_ strains, natural transformation was conducted on solid medium at 37°C as follows. The strains were streaked on CYE solid medium from a frozen stock culture and incubated for 72 h at 37°C. The strains were then restreaked on a new CYE plate and incubated overnight at 37°C to obtain freshly growing cells. Bacteria were resuspended in sterile water to an OD_600_ of 1 to obtain a suspension of 1.10^9^ CFU/ml. A 10-μl portion of the suspensions (∼1 × 10^7^ CFU) was spotted on CYE with 1.5 μg of transforming DNA. Once the spots were absorbed by the agar, plates were incubated at 37°C for 24 h. Each spot was resuspended in 200 μl sterile water and used to perform 10-fold serial dilutions, which were then plated on nonselective medium and selective medium. Plates were incubated at 37°C for 72 h. Finally, transformation frequencies were calculated as the ratio of the number of CFU counted on selective medium divided by the number of CFU counted on nonselective medium. For all the *rocC*_TAA_ strains, the *rpsL* PCR product was used as transforming DNA. This transforming DNA is obtained by amplifying the 2-kb regions upstream and downstream of the *rpsL* single point mutation conferring resistance to streptomycin (PCR primer pair rpsL_F/rpsL_R). Transformation experiments on strains bearing plasmids p0681F and p1890F were performed the same way, using CYE plates containing different concentrations of IPTG.

For the non-constitutively transformable strains of L. pneumophila, transformation was carried out in liquid medium at 30°C as follows. Strains were streaked on CYE solid medium from a frozen stock culture, incubated for 72 h at 37°C, restreaked on a new CYE plate, and incubated overnight at 37°C. Fresh bacteria were resuspended in 3 ml of AYE in 13-ml tubes to an OD of ∼0.2 with 2 μg of transforming DNA and cultivated at 30°C with shaking for 24 h. Tenfold serial dilutions of each culture were then performed, plated on nonselective medium and selective medium, and incubated at 37°C for 72 h. Finally, transformation frequencies were determined as described above. (3) For *letA* mutants of constitutively and non-constitutively transformable strains of L. pneumophila: strains were streaked on CYE solid medium from a frozen stock culture, incubated for 72 h at 37°C, restreaked on a new CYE plate, and incubated overnight at 37°C. Fresh bacteria were resuspended in 3 ml of AYE in 13-ml tubes to an OD of ∼0.2 and cultivated at 30°C with shaking until an OD of ∼2 to 4 (corresponding to the competence phase of L. pneumophila) was reached. A volume corresponding to 1 × 10^8^ bacteria was spotted on CYE plates with 1.5 μg of the *rpsL* PCR product. The following steps were the same as for the transformation of constitutively transformable *rocC*_TAA_ strains as mentioned in item 1 above.

### Detection of extracellular pilin by Western blotting.

Strains bearing plasmid p1890F were grown overnight at 37°C on CYE containing 500 μM IPTG and were then resuspended in 2 ml AYE at an optical density at 600 nm (OD_600_) of ∼1.5. One milliliter of the suspension was then subjected to maximum-speed vortex agitation for 1 min and centrifuged for 15 min at 21,000 × *g* and 4°C. Supernatants were recovered in a new tube and centrifuged again, while pellets were saved on ice. After centrifugation, 900 μl of supernatants were recovered and proteins were precipitated by adding 100 μl of trichloroacetic acid (TCA; final concentration of 10%). After 30 min of incubation on ice, a 15-min centrifugation at 21,000 × *g* and 4°C was performed. Pellets were washed three times with acetone, dried at room temperature, and resuspended with 100 μl of 1× Laemmli sample buffer. Pellets previously saved on ice were resuspended with 150 μl of 1× sample buffer. Samples were then analyzed by Western blotting. Aliquots were boiled for 5 min and subjected to denaturing polyacrylamide gel electrophoresis. Proteins from SDS-polyacrylamide gels were electrophoretically transferred to nitrocellulose membranes (Schleicher and Schuell) and subsequently stained with Ponceau S (Sigma) to check the loading of the lanes. Membranes were incubated with monoclonal anti-FLAG antibody (1:1,000 dilution; no. F1804; Sigma) as a primary antibody and an anti-mouse immunoglobulin–peroxidase conjugate (1:50,000 dilution; no. A0168; Sigma) as a secondary antibody. Nitrocellulose membranes were revealed with the SuperSignal West Dura detection system (Pierce) and an imaging workstation equipped with a charge-coupled device camera (Thermo).

### Determination of the DNA uptake ability.

The ability of the transformation-deficient mutants to take up DNA was determined as follows: strains were inoculated in AYE medium at an OD_600_ of 0.05, and tubes were incubated overnight under constant shaking at 30°C. When an OD_600_ of 0.9 was reached, 1 ml of each culture was centrifuged for 3 min at 5,000 × g, and pellets were resuspended in 200 μl ultrapure water containing 2 μg of pGEM-HYG1. This plasmid is a nonreplicative plasmid in L. pneumophila and, as it contains no homology with the L. pneumophila genome, it cannot integrate by recombination. After 20 min of incubation at 37°C, tubes were centrifuged for 3 min at 5,000 × *g*, and pellets were resuspended in 200 μl AYE liquid medium containing 10 U of DNase I (Sigma). After 20 min of incubation at 37°C, DNase I was removed, and bacteria were washed by two successive centrifugations for 3 min at 5,000 × *g* and resuspension in 1 ml of water. Pellets were finally resuspended in 100 μl ultrapure water and incubated for 30 min at 65°C to complete DNase I inactivation and kill bacteria. The DNA uptake ability of each mutants was then determined by PCR, using two pairs of primers amplifying on the one hand the chromosomal *mreB* gene (*lpp0873*) and on the other hand a part of pGEM-HYG1, giving, respectively, PCR products of 1,194 bp (mreBseqF/mreBseqR) and 1,657 bp [M13F(-47)/M13R(-48)].

### Microscopy.

Bacteria expressing the FLAG-tagged pilins were grown as spots on CYE plates with 0.5 mM IPTG for 24 h at 37°C. Bacteria were gently collected with a pipette tip. In order to limit shearing and breaking of the pilus, the pipette tip was left standing an Eppendorf tube with 1 ml of water for a few minutes. Once the collected bacterial culture was starting to dissociate and falling off the tip, the bacterial pellet was resuspended gently by slowly pipetting up and down. The collected 1-ml suspensions were centrifuged for 3 min at 5000 × *g*, and pellets were gently resuspended in 300 μl PBS–3.7% formaldehyde and incubated at room temperature for 30 min. Acid-washed (ethanol-HCl, 1 M) glass coverslips were coated with poly-l-lysine by immersion in a 0.01% poly-l-lysine solution in distilled water (Sigma-Aldrich) for 5 min. Fixed bacteria in PBS–3.7% formaldehyde were pipetted (250 μl) on the air-dried coverslips and allowed to settle and stick to the coverslips for about 30 min. Coverslips were then washed twice with PBS and incubated with monoclonal anti-FLAG M2–fluorescein conjugates at 1/200 in PBS for 1 h. Coverslips were then washed twice with PBS, and DNA was labeled using Hoechst 33288 (12 μg/ml in PBS) for 1 h. Coverslips were washed twice in PBS and mounted using 8 μl of mounting solution (1,4-diazabicyclo[2.2.2]-octane [DAPCO]). After an overnight incubation at 4°C, slides were observed and imaged with an epifluorescence microscope (Zeiss Axioplan 2).

### Genome sequencing.

The complete genome of isolate HL-0709-3014 was obtained using Illumina MiSeq paired-end reads from previously available SRA sample ERS1305867 and long reads from Oxford Nanopore sequencing on a MinION sequencer according to the manufacturer’s instructions (Oxford Nanopore). Illumina and Nanopore reads were then used for short-read/long-read hybrid assembly using Unicycler v0.4.6 (87).

### Data availability.

The complete genome of isolate HL-0709-3014 is available under accession numbers CP048618.1 (chromosome) and CP048619.1 (plasmid). The strain is listed under the name Legionella pneumophila strain ERS1305867 (BioProject no. PRJEB15241; BioSample no. SAMEA4394418). Raw sequencing reads of the Tn-seq samples are available at the European Nucleotide Archive (https://www.ebi.ac.uk/ena/) under the study accession number PRJEB40244.

## Supplementary Material

Supplemental file 1

Supplemental file 2
