# Supplementary material for: Transposon Insertion Sequencing in a Clinical Isolate of Legionella pneumophila Identifies Essential Genes and Determinants of Natural Transformation
Source: J Bacteriol. 2021 Jan 11;203(3):e00548-20. doi: 10.1128/JB.00548-20 (PMC7811196; doi:10.1128/JB.00548-20)
Supplement: Supplemental file 1 [file JB.00548-20-s0001.pdf]

Transposon-insertion sequencing in a clinical isolate of *Legionella pneumophila* identifies essential genes and determinants of natural transformation

Léo Hardy, Pierre-Alexandre Juan, Bénédicte Coupat-Goutaland and Xavier Charpentier

Supplemental material

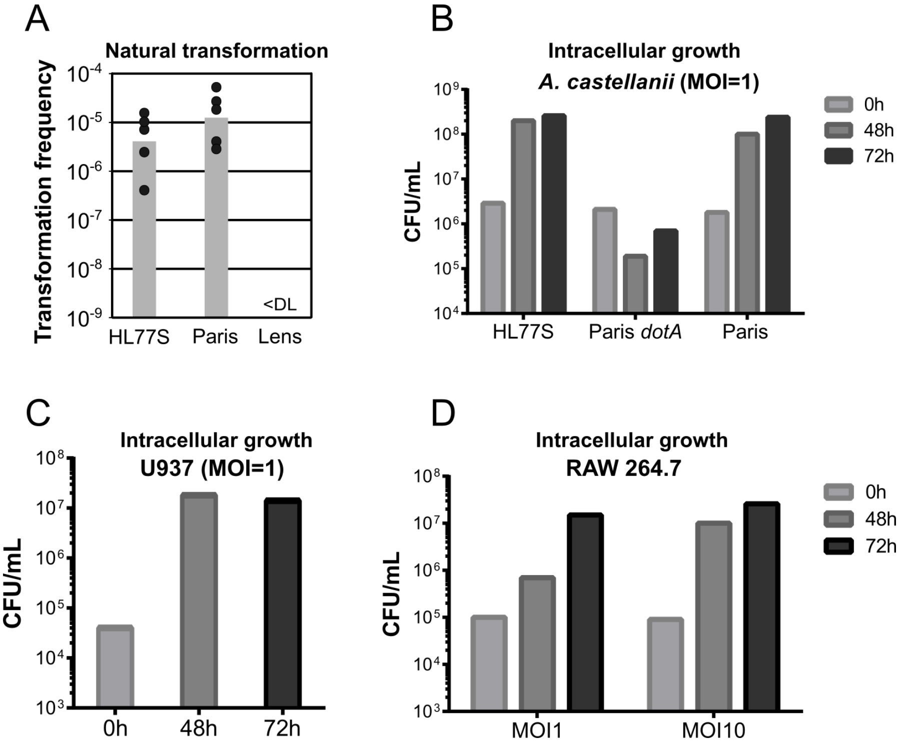

Figure S1. Phenotypic characterization of strain HL77S, a streptomycin-resistant mutant of the clinical isolate HL-0709-3014.

(A) Natural transformability of HL77S compared to the Paris and Lens strain. Transformation was tested by growing the strains in AYE at 30°C for 24h in the presence of 2 µg of transformation DNA consisting of a kanamycin resistance gene interrupting the ihfB gene. (B) Intracellular replication of HL77S in the amoeba *A. castellanii*. Cells were infected with a suspension of HL77S at a multiplicity of infection (MOI) of 1. At the initial time point, after 48h and 72h of culture at 30°C, colony-forming units are determined by plating on CYE medium. (C) Intracellular replication of HL77S in differentiated human monocytes of the U937 cell line. Cells were infected with a suspension of HL77S at a multiplicity of infection (MOI) of 1. At the initial time point, after 48h and 72h of culture at 30°C, colony-forming units are determined by plating on CYE. (D) Intracellular replication of HL77S in the murine macrophage-like cell line RAW 264.7. Cells were infected with a suspension of HL77S at a multiplicity of infection (MOI) of 1 or 10. At the initial time point, after 48h and 72h of culture at 30°C, colony-forming units are determined by plating on CYE. Data from panel A, B and C are representative of experiment performed at least three times.

Dataset S1. Tn-seq data for strain HL77S in Excel spreadsheet. Spreadsheet “Analysis – Summary” lists gene annotations and gene essentiality on 1st and 2nd isolation (NE, non-essential; E, essential; U, uncertain; GA, growth advantage; GD, growth disadvantage; S, too short), gene fitness value (log_2 fold-change 2nd vs 1st isolation) and gene role in natural transformation by the *ihfB*::kan and *legK2*::kan markers (log_2 fold-change between transformed and control populations). Following spreadsheets includes all raw data for the analysis displayed in the summary. Spreadsheet “Sample description” provides description of the samples and read mapping statistics.

Table S1. Strains, plasmids and oligonucleotides used in this study

Strains

Name	Genotype	Source
Paris WT	Wild-type *Legionella pneumophila*	Paris Outbreak isolate CIP107629, CNR Lyon
Paris_S	Spontaneous streptomycin-resistant mutant of Paris; StrepR	This study
HL-0709-3014	Wild-type *Legionella pneumophila*	Clinical isolate, CNR Lyon
HL77S	Spontaneous streptomycin-resistant mutant of HL-0709-3014; StrepR	This study
Paris *rocC*$_{TAA}$	Paris *rocC*$_{TAA}$ *(=lpp0148*$_{TAA}$*); rocC* allele with a premature stop codon TAA	(1)
Paris *rocC*$_{TAA}$ *letS::kan*	Paris *rocC*$_{TAA}$*; lpp1887::kan; KanR*	This study
Paris *rocC*$_{TAA}$ *letA::kan*	Paris *rocC*$_{TAA}$*; lpp2699::kan; KanR*	This study
Paris *rocC*$_{TAA}$ *comM::kan*	Paris *rocC*$_{TAA}$*; lpp2632::kan; KanR*	This study
Paris *rocC*$_{TAA}$ *comEC::kan*	Paris *rocC*$_{TAA}$*; lpp0680::kan; KanR*	This study
Paris *rocC*$_{TAA}$ *pilE::kan*	Paris *rocC*$_{TAA}$*; lpp0681::kan; KanR*	This study
Paris *rocC*$_{TAA}$ *comEA::kan*	Paris *rocC*$_{TAA}$*; lpp0872::kan; KanR*	This study
Paris *rocC*$_{TAA}$ *pilA1::kan*	Paris *rocC*$_{TAA}$*; lpp1890::kan; KanR*	This study
Paris *rocC*$_{TAA}$ *lpp1976-1977-1978::kan*	Paris *rocC*$_{TAA}$*; lpp1976-1977-1978::kan; KanR*	This study
Paris *rocC*$_{TAA}$ *comF::kan*	Paris *rocCTAA; lpp2280::kan; KanR*	This study
Paris *rocC*$_{TAA}$ *pilA2::kan*	Paris *rocC*$_{TAA}$*; lpp1889::kan; KanR*	This study
Paris *rocC*$_{TAA}$ *pilZ::kan*	Paris *rocC*$_{TAA}$*; lpp1356::kan; KanR*	This study
Paris *rocC*$_{TAA}$ *lpp3030::kan*	Paris *rocC*$_{TAA}$*; lpp3030::kan; KanR*	This study
Paris *rocC*$_{TAA}$ *djlA::kan*	Paris *rocC*$_{TAA}$*; lpp2289::kan; KanR*	This study
Paris *rocC*$_{TAA}$ *lpp2632::kan*	Paris *rocC*$_{TAA}$*; lpp2632::kan; KanR*	This study
Paris *letA::kan*	Paris *lpp2699::kan; KanR*	This study
Paris *dotA::kan*	Paris *dotA::kan; KanR*	This study
MFDpir	*Escherichia coli*; MG1655 RP4-2-Tc::[ΔMu1::aac(3)IV-ΔaphA-Δnic35-ΔMu2::zeo] ΔdapA::(erm-pir) ΔrecA	(2)

Plasmids

Name	Genotype	Description	source
pGEM-	pGEM-T Easy::	plasmid bearing the *ihfB* gene of *L. pneumophila*	(1)

ihfB::kan	*ihfB*::*nptII*; AmpR, KanR	Paris interrupted by a kanamycin cassette ; Kan R	
pGEM-HYG1	pGEM-T Easy::*hygR*	plasmid bearing an hygromycine cassette ; HygR	This study
pJET1.2-legk2::kan	pJET1.2 ; *legk2*::kan from HL77 ; AmpR, KanR	plasmid bearing the *legK2* gene of L. pneumophila HL77 interrupted by a kanamycin cassette ; Kan R	This study
pBT20	Gmr; Himar1C9 transposon	Pir-dependent, mobilizable plasmid carrying the Mariner Himar1 transposon with gentamycin resistant gene	(3)
pMMB207C	RSF1010::$lacI^q$-*tacp mobA*	cloning vector for expression genes under the Ptac promoter, derived from RSF1010 ; CmR	(4)
p0681F	pMMB207C ; lpp0681::FLAG	complementation plasmid for the lpp0681::kan mutant, expressing the lpp0681-FLAG (PilE-FLAG) fusion protein ; CmR	This study
p1890F	pMMB207C ; lpp1890::FLAG	complementation plasmid for the lpp1890::kan mutant, expressing the lpp1890-FLAG (pilA2-FLAG) fusion protein ; CmR	This study

Oligonucleotides

**Name**	**sequence**	**use**
LH1_lpp3030_P1	ctgcatgggatattggtatcactgc	Forward primer to amplify a 2kB fragment uptstream of lpp3030
LH2_lpp3030_P2-tail-pKD4	GGAACTTCGAAGCAGCTCCAGCCTAC ACAATCtggcgatgataaacaagcgagagcc	Reverse primer to amplify a 2kB fragment uptstream of lpp3030
LH3_lpp3030_P3-tail-pKD4	GAACTAAGGAGGATATTCATATGGACC ATGGCgaaaatctgccgatactgtttctgc	Forward primer to amplify a 2kB fragment downstream of lpp3030
LH4_lpp3030_P4	ggtcccatctcattctccttaatcc	Reverse primer to amplify a 2kB fragment downstream of lpp3030
LH5_lpp1887_P1	gggtaatttcctgagacagtggagg	Forward primer to amplify a 2kB fragment uptstream of letS
LH6_lpp1887_P2-tail-pKD4	GGAACTTCGAAGCAGCTCCAGCCTAC ACAATCggcaggaataagagtagtgattcttagc	Reverse primer to amplify a 2kB fragment uptstream of letS
LH7_lpp1887_P3-tail-pKD4	GAACTAAGGAGGATATTCATATGGACC ATGGCctaatgctgataatttaaccggagcc	Forward primer to amplify a 2kB fragment downstream of letS
LH8_lpp1887_P4	ggtggaggtaccatagttatgaccc	Reverse primer to amplify a 2kB fragment downstream of letS
LH9_lpp2699_P1	ggtatcctgtctgacagtctaaacc	Forward primer to amplify a 2kB fragment uptstream of letA
LH10_lpp2699_P2-tail-pKD4	GGAACTTCGAAGCAGCTCCAGCCTAC ACAATCtcccattctaaccaatgcatggtc	Reverse primer to amplify a 2kB fragment uptstream of letA
LH11_lpp2699_P3-tail-pKD4	GAACTAAGGAGGATATTCATATGGACC ATGGCcgcattattgaacaccccaatg	Forward primer to amplify a 2kB fragment downstream of letA
LH12_lpp2699_P4	caggggaaatcaaagacattgcc	Reverse primer to amplify a 2kB fragment downstream of letA
LH21_lpp2632_P1	cggcttcttgatattattgcgagac	Forward primer to amplify a 2kB fragment uptstream of lpp2632
LH22_lpp2632_P2-tail-pKD4	GGAACTTCGAAGCAGCTCCAGCCTAC ACAATCcgagacacactatctcgtatcatgc	Reverse primer to amplify a 2kB fragment uptstream of lpp2632
LH23_lpp2632_P3-tail-pKD4	GAACTAAGGAGGATATTCATATGGACC ATGGCgtgcatactttggtcttgggaagac	Forward primer to amplify a 2kB fragment downstream of lpp2632
LH24_lpp2632_P4	gatggagaaatttttccgctcatcc	Reverse primer to amplify a 2kB fragment downstream of lpp2632
LH29_lpp2289_P1	caggaatgttcacactgaattttcc	Forward primer to amplify a 2kB fragment uptstream of djlA
LH30_lpp2289_P2-tail-pKD4	GGAACTTCGAAGCAGCTCCAGCCTAC ACAATCcccccaccaggttgttattacaaag	Reverse primer to amplify a 2kB fragment uptstream of djlA
LH31_lpp2289_P3-tail-pKD4	GAACTAAGGAGGATATTCATATGGACC ATGGCgctcaaggtttacccgaagaaatg	Forward primer to amplify a 2kB fragment downstream of djlA
LH32_lpp2289_P4	cgagtcagggaagtgtttacagg	Reverse primer to amplify a 2kB fragment downstream of djlA

rpsL_Fw	GCAGCTCCAGATGGCTCAATC	Forward primer to amplify a 2kB fragment downstream of rpsL
rpsL_Rv	CAACCATACATGTCCATATTGACCAC	Reverse primer to amplify a 2kB fragment uptstream of rpsL
lpp0640_P1	TCATCCAACTCATCTCGCAATCG	Forward primer to amplify a 2kB fragment uptstream of comM
lpp0640_P2-tail-pKD4	GGAACTTCGAAGCAGCTCCAGCCTACACAATCACTGCTGTTTCAGCAAGTCCTACC	Reverse primer to amplify a 2kB fragment uptstream of comM
lpp0640_P3-tail-pKD4	GAACTAAGGAGGATATTCATATGGACCATGGCGTCTGCTCGTGGCTACCATCGC	Forward primer to amplify a 2kB fragment downstream of comM
lpp0640_P4	GCTGTAGGACAGCGGCTAACTTG	Reverse primer to amplify a 2kB fragment downstream of comM
lpp0681_P1	ATGGGAGCTGGCGTAGATCCTG	Reverse primer to amplify a 2kB fragment downstream of pilE
lpp0681_P2-tail-pKD4	GGAACTTCGAAGCAGCTCCAGCCTACACAATCGGCAATTGAAACCAGAATGCCC	Reverse primer to amplify a 2kB fragment downstream of pilE
lpp0681_P3-tail-pKD4	GAACTAAGGAGGATATTCATATGGACCATGGCAAACAACGCCGAACGGGTAC	Reverse primer to amplify a 2kB fragment downstream of pilE
lpp0681_P4	CATTGCCATTGCGGGTATGAATG	Reverse primer to amplify a 2kB fragment downstream of pilE
lpp1889_P1	GTGAACTGCAGCAAGCTCCATCC	Reverse primer to amplify a 2kB fragment downstream of pilA1
lpp1889_P2-tail-pKD4	GGAACTTCGAAGCAGCTCCAGCCTACACAATCGATGGCAACCAGAATCCCAAG	Reverse primer to amplify a 2kB fragment downstream of pilA1
lpp1889_P3-tail-pKD4	GAACTAAGGAGGATATTCATATGGACCATGGCTGCAAACGCAGGTAATGGCAC	Reverse primer to amplify a 2kB fragment downstream of pilA1
lpp1889_P4	GCGAAATGGCCGTTACTGCTTG	Reverse primer to amplify a 2kB fragment downstream of pilA1
lpp1890_P1	GTCAGGTAATAACCGGGTTTGCC	Reverse primer to amplify a 2kB fragment downstream of pilA2
lpp1890_P2-tail-pKD4	GGAACTTCGAAGCAGCTCCAGCCTACACAATCGCGATTGCTGCCAAAATACCG	Reverse primer to amplify a 2kB fragment downstream of pilA2
lpp1890_P3-tail-pKD4	GAACTAAGGAGGATATTCATATGGACCATGGCAATTGCTATTGGTGCGAACGG	Reverse primer to amplify a 2kB fragment downstream of pilA2
lpp1890_P4	CAGTCTGGTGGTGTGACCGCTG	Reverse primer to amplify a 2kB fragment downstream of pilA2
lpp2280_P1	CCGGCAACTGGAAAAGGGAG	Reverse primer to amplify a 2kB fragment downstream of comF
lpp2280_P2-tail-pKD4	GGAACTTCGAAGCAGCTCCAGCCTACACAATCTCCATGCAATTAGAGCAAACTGC	Reverse primer to amplify a 2kB fragment downstream of comF
lpp2280_P3-tail-pKD4	GAACTAAGGAGGATATTCATATGGACCATGGCAAGCCAACCTTGATGGAGAGC	Reverse primer to amplify a 2kB fragment downstream of comF
lpp2280_P4	GCCCGCAAGCTACAAATACCATAG	Reverse primer to amplify a 2kB fragment downstream of comF
lpp1976-1978_P1	CAATCAATCAAACTCTCTCAAGAACG	Reverse primer to amplify a 2kB fragment downstream of the operon of unknown function
lpp1976-1978_P2-tail-pKD4	GGAACTTCGAAGCAGCTCCAGCCTACACAATCGCCAGATAAGATGATTGATTAGATCTC	Reverse primer to amplify a 2kB fragment downstream of the operon of unknown function
lpp1976-1978_P3-tail-pKD4	GAACTAAGGAGGATATTCATATGGACCATGGCGGTGAAGGAAGGGGTAAGCAGC	Reverse primer to amplify a 2kB fragment downstream of the operon of unknown function
lpp1976-1978_P4	GAAAAATGAATGGGAGCTTCTGG	Reverse primer to amplify a 2kB fragment downstream of the operon of unknown function
lpp0872_P1	GTAGTGGGAGGAAGCTTAGG	Reverse primer to amplify a 2kB fragment downstream of comEA
lpp0872_P2-tail-pKD4	GGAACTTCGAAGCAGCTCCAGCCTAC	Reverse primer to amplify a 2kB

	ACAATCGAGGCTCGTTCTCAGCTTGAGAAGAG	fragment downstream of comEA
lpp0872_P3-tail-pKD4	GAACTAAGGAGGATATTCATATGGACCATGGCGCGGAAGTCAAGGGTATAGG	Reverse primer to amplify a 2kB fragment downstream of comEA
lpp0872_P4	GCGACAGGGCTAACTGTAAC	Reverse primer to amplify a 2kB fragment downstream of comEA
olj376	GTGACTGGAGTTCAGACGTGTGCTCTTCCGATCTGGGGGGGGGGGGGGGG	Primer for Tn-seq to amplify transposon junction and add polyC-tail during PCR1
pBT20-PCR1	Biotin-TCGTATAATGTGTGGAATTGTGAGCGG	Biotinylated primer for Tn-seq to amplify transposon junction during PCR1
pBT20-PCR2	AATGATACGGCGACCACCGAGATCTACACTCTTTGGACTCTAGAGGATCACCCAGCTTTCTTG	Primer for Tn-seq to amplify transposon junction during PCR2
TdT_Index_X	CAAGCAGAAGACGGCATACGAGATXXXXXXGTGACTGGAGTTCAGACGTGTGCTCTTCCGATCT	HPLC-purified primer for Tn-seq to amplify transposon junction and add index for multiplexing during PCR2
Read1TnLp	CTAGAGACCGGGGACTTATCAGCCAACCTGTTA	HPLC-purified custom sequencing primer for Tn-seq library sequencing
lpp0681-F	CCGGGGATCCGCAAATTCAATAGAGGATACCCAAATG	cloning of lpp0681 in pMM207C
lpp0681F-R	AGCCAAGCTTTTACTTGTCATCGTCGTCCTTGTAATCAGCGCTACCGGGATTCCAGCATTCTGGTTG	cloning of lpp0681 in pMM207C
lpp1890-F	CCGGGGATCCGTTAACTATGGAGATGGTCATGAGAC	cloning of lpp1890 in pMM207C
lpp1890F-R	AGCCAAGCTTTTACTTGTCATCGTCGTCCTTGTAATCAGCGCTTGGTCTGCAGCTGGCAGGTCG	cloning of lpp1890 in pMM207C
mreBseqF	TCCGGTTAAAAGCAGTTGTCTGG	Forward primer to amplify *mreB*, chromosomal control for uptake test
mreBseqR	CCCAGAGAACTGTGTCCGCCC	Reverse primer to amplify *mreB*, chromosomal control for uptake test
M13F(-47)	CGCCAGGGTTTTCCCAGTCACGAC	Forward primer to amplify a 1657 pb fragment of the pGEM-HYG1
M13R(-48)	AGCGGATAACAATTTCACACAGGA	Reverse primer to amplify a 1657 pb fragment of the pGEM-HYG1

References

1. Juan P-A, Attaiech L, Charpentier X. 2015. Natural transformation occurs independently of the essential actin-like MreB cytoskeleton in Legionella pneumophila. Sci Rep 5:16033.

2. Ferrières L, Hémery G, Nham T, Guérout A-M, Mazel D, Beloin C, Ghigo J-M. 2010. Silent Mischief: Bacteriophage Mu Insertions Contaminate Products of Escherichia coli Random Mutagenesis Performed Using Suicidal Transposon Delivery Plasmids Mobilized by Broad-Host-Range RP4 Conjugative Machinery. J Bacteriol 192:6418–6427.

3. Kulasekara HD, Ventre I, Kulasekara BR, Lazdunski A, Filloux A, Lory S. 2005. A novel two-component system controls the expression of Pseudomonas aeruginosa fimbrial cup genes. Mol Microbiol 55:368–380.

4. Segal G, Shuman HA. 1998. Intracellular multiplication and human macrophage killing by Legionella pneumophila are inhibited by conjugal components of IncQ plasmid RSF1010. Mol Microbiol 30:197–208.
